# AI-Powered Mobile App for Nuclear Cataract Detection

**DOI:** 10.3390/s25133954

**Published:** 2025-06-25

**Authors:** Alicja Anna Ignatowicz, Tomasz Marciniak, Elżbieta Marciniak

**Affiliations:** 1 Division of Electronic Systems and Signal Processing, Institute of Automatic Control and Robotics, Poznan University of Technology, 60-965 Poznan, Poland; alicja.ignatowicz@student.put.poznan.pl; 2 Department of Ophthalmology, Chair of Ophthalmology and Optometry, Heliodor Swiecicki University Hospital, Poznan University of Medical Sciences, 60-780 Poznan, Poland; emarciniak@ump.edu.pl

**Keywords:** cataract, neural networks, smartphone app, Android

## Abstract

Cataract remains the leading cause of blindness worldwide, and the number of individuals affected by this condition is expected to rise significantly due to global population ageing. Early diagnosis is crucial, as delayed treatment may result in irreversible vision loss. This study explores and presents a mobile application for Android devices designed for the detection of cataracts using deep learning models. The proposed solution utilizes a multi-stage classification approach to analyze ocular images acquired with a slit lamp, sourced from the Nuclear Cataract Database for Biomedical and Machine Learning Applications. The process involves identifying pathological features and assessing the severity of the detected condition, enabling comprehensive characterization of the NC (nuclear cataract) of cataract progression based on the LOCS III scale classification. The evaluation included a range of convolutional neural network architectures, from larger models like VGG16 and ResNet50, to lighter alternatives such as VGG11, ResNet18, MobileNetV2, and EfficientNet-B0. All models demonstrated comparable performance, with classification accuracies exceeding 91–94.5%. The trained models were optimized for mobile deployment, enabling real-time analysis of eye images captured with the device camera or selected from local storage. The presented mobile application, trained and validated on authentic clinician-labeled pictures, represents a significant advancement over existing mobile tools. The preliminary evaluations demonstrated a high accuracy in cataract detection and severity grading. These results confirm the approach is feasible and will serve as the foundation for ongoing development and extensions.

## 1. Introduction

Cataract is an eye disease that causes the lens of the eye to become cloudy, which leads to decreased visual acuity. Cataract caused 39.55% (95% uncertainty interval (UI): 33.48, 46.34%) of all blindness in 2020 worldwide [1]. It is the most common cause of vision loss, especially among older people, due to the natural ageing process of the human eye. Other causes of cataracts include genetic predisposition (congenital cataracts), long-term use of steroids, and smoking [2].

Age-related cataract typically leads to a progressive decline in visual acuity, which, in its advanced stages, can culminate in complete vision loss. Statistical analyses have indicated that the global prevalence is estimated at 17.2%, with nuclear cataracts being the most common subtype. The study found significant regional differences, with higher rates in Africa and Southeast Asia and lower rates in Europe and North America. Prevalence increased markedly with age, exceeding 30% in people over 70 years old [3]. However, the distribution of this condition differs across various geographic regions. This variation likely stems from differences in ultraviolet radiation exposure, nutritional status, lifestyle choices, and the availability and quality of eye care services, all of which significantly impact eye health.

Treatment for cataracts is based on the patient’s visual acuity. If visual acuity is 6/24 or better, corrective eyeglasses or eye drops for pupil dilation—such as 2.5% phenylephrine or atropine—may suffice. Conversely, if visual acuity falls below 6/24 or if there are other medical concerns, surgical options become necessary. For senile cataracts, the surgical methods available include extracapsular cataract extraction (ECCE), intracapsular cataract extraction (ICCE), phacoemulsification (most common method), or laser [4].

### 1.1. Cataract Types

There are various types of cataracts, classified according to their epidemiological characteristics. The most prevalent form of cataract is senile cataract, affecting the elderly population. It is marked by a gradual opacification of the crystalline lens, primarily due to age-related oxidative stress. With advancing age, the lens’ ability to detoxify reactive oxygen species (ROS) diminishes, leading to a buildup of nonenzymatic modifications in crystallin proteins. These biochemical changes result in protein denaturation and lipid peroxidation, forming high-molecular-weight complexes [3]. Consequently, this increases light scattering within the lens, compromising its transparency.

**Figure 1:**
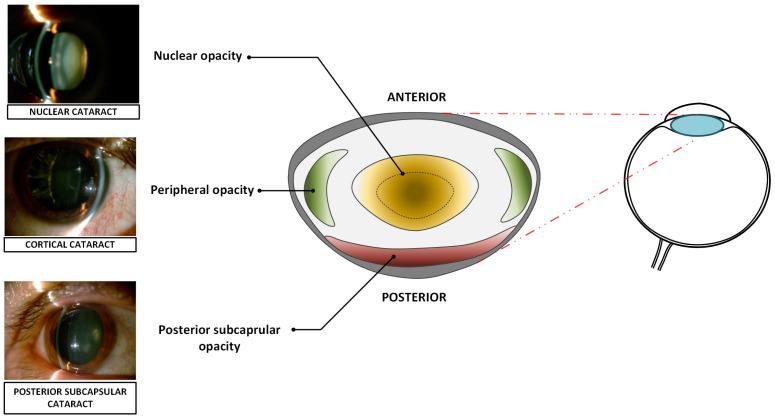
Anatomical localization of various senile cataracts within the human crystalline lens.

Morphologically, senile cataract is categorized into distinct subtypes, each exhibiting specific localization and predominant pathological mechanisms, as presented on Figure 1:

- Nuclear cataract,
- Cortical cataract,
- Posterior subcapsular cataract.

In nuclear cataract, oxidative damage leads to the denaturation and aggregation of crystallin proteins in the central region of the lens, resulting in nuclear sclerosis and progressive discoloration, ranging from yellowing to brunescence [5].

Cortical cataract is characterized by spoke-like, wedge-shaped opacities originating in the peripheral cortex of the lens and progressing toward the center. These opacities may involve the anterior, equatorial, or posterior cortical layers. Patients with cortical cataracts frequently report photophobia and glare [6].

Posterior subcapsular cataract (PSC) is a form of cataract characterized by opacification located directly beneath the posterior capsule of the lens, within the visual axis. This results in significant impairment of visual acuity, particularly under bright lighting conditions, and causes difficulties with near tasks such as reading. PSC often progresses more rapidly than other types of cataracts [7,8].

There are also other types of cataracts, e.g., congenital cataracts that can be present at birth or diagnosed within the first year of life. If not identified and treated promptly, these can cause irreversible visual impairment. Cases of this type of cataract are associated with genetic factors or intrauterine infections caused by viruses. The most characteristic clinical signs include leukocoria (a white pupillary reflex) and strabismus [9].

### 1.2. Related Work

Modern smartphones, which have high computing power, advanced cameras, and touch screens for easy image segmentation open up new possibilities for so-called telemedicine. A number of mobile application solutions are also intended for use in ophthalmology. Most available ophthalmology-related applications focus on raising awareness, educating users [10,11], checking visual acuity [12,13], or making predictions based on entered numerical parameters [14]. They can be used by patients with AMD, diabetic retinopathy, glaucoma, or color vision deficiency. Among the solutions analyzed in a systematic review article [15], the authors indicated in the statistics that three solutions concerned cataracts, although the descriptions only included information about the “CRADLE White Eye Detector” application, which enables the detection of leucoria [16]. Of course, there are solutions available in the literature directly related to cataracts and an overview of these is presented below.

Already in 2015, the authors of the article in [17] presented the concept of a low-cost solution for detecting cataracts. The proposed system assumed image acquisition using a smartphone with an external microscopic lens. Only a concept was presented, without implementation details and without tests verifying the accuracy of detection.

A low-cost solution was also the subject of the publication in [18], in which the authors presented the possibility of preparing an application using Android Application Software for detecting cataract with a running time camera. It should be noted that only a general pseudocode was presented, and the detection itself was to be based on simple color thresholding. Similarly to the previous article, there were no tests verifying the effectiveness of the solution.

A more detailed description of a solution for detecting and classifying nuclear cataracts based on slit lamp images can be found in [19], where the authors used artificial intelligence techniques, including localization using YOLO v3 and classification using ShuffleNet and SVM. The solution, named UFDA (Unified Diagnosis Framework for Automated Nuclear Cataract Grading), according to the authors, achieved an accuracy of 93.48%. The images were acquired using a smartphone-based slit lamp, but no detailed information about the device was provided. In addition, the Marked Slit Lamp Picture Project (MSLPP) dataset used in the experiments is not publicly available.

Machine learning techniques, specifically SVM (support vector machines) were used in the solution presented in [20], where the authors considered using iPhone-type smartphones for the cataract detection process. The obtained accuracy was as high as 96.6%, but it should be noted that photos of an eye model (interestingly, many times larger than the human eye) were used for training and testing, not real images.

Research on cataract detection using a smartphone and taking into account computational efficiency limitations can be found in [21], which proposed an Optimised Light Weight Sequential Deep Learning Model (SDLM) and an application prepared using Android Studio. The presented convolutional neural network model has 13 layers and, according to the authors, had an accuracy of 93.44%. Tests were conducted using data from Kaggle and personally gathered. Unfortunately, neither the data nor the software were made available.

Summarizing the analysis of publications on cataract recognition using a smartphone, it can be seen that it is difficult to reproduce the experiments described in the above articles, and the authors do not provide data and software. Our solution is free from such limitations and allows the reader to further modify and experiment. The main contributions of the study presented in this article are as follows:

- a comparison of the effectiveness of six neural network architectures trained using real slit-lamp photos;
- a comprehensive comparison of the effectiveness neural network models after conversion to the constraints of mobile devices;
- full source code of a mobile app designed for cataract classification, with software released on GitHub (version 1.0).

## 2. Materials and Methods

As the use of mobile diagnostic tools increases, their development increasingly relies on publicly accessible datasets for eye diseases, which aid in training and testing detection algorithms. However, despite the growing interest in AI-based ophthalmologic solutions, there is still a limited number of publicly available datasets specifically focused on cataracts. The next subsection briefly describes the datasets that appear in scientific publications.

### 2.1. Publicly Available Datasets

Several publicly available datasets include video recordings of cataract surgeries. The Cataract-1K dataset [22] comprises 1000 videos of cataract removal procedures performed between 2021 and 2023, with an average duration of 7.12 min. The recordings were captured using a MediLive Trio Eye device mounted on a ZEISS OPMI Vario microscope (Carl Zeiss Meditec AG, Jena, Germany).

The CATARACTS dataset [23] includes 50 high-resolution videos (1920×1080 pixels) of cataract extraction procedures. Each surgery was recorded from two perspectives: a microscope view (30 frames per second) and a surgical tray view (50 frames per second), with an average duration of 10 min and 56 s, recorded using a 180I camera (Toshiba, Tokyo, Japan) mounted on an OPMI Lumera T surgical microscope (Carl Zeiss Meditec AG, Jena, Germany).

There are datasets that include slit-lamp cataract images used for clinical studies and research. The Nuclear Cataract Database for Biomedical and Machine Learning Applications [24] contains 1437 slit-lamp images classified by ophthalmologists using the LOCS III (Lens Opacities Classification System III) [25]. This established grading system evaluates cataract severity based on four criteria: NC (nuclear color), NO (nuclear opalescence), C (cortical opacities), and P (posterior subcapsular opacities). Each criterion is scored on a continuous scale from 0.1 to 6.9, with higher scores indicating greater severity of opacification. Images were acquired using a Topcon DC-1 digital slit-lamp camera and a Topcon DC-3 digital slit-lamp camera, mounted respectively on Topcon SL-D2 and SL-D7 slit-lamp bases (Topcon Corporation, Tokyo, Japan).

### 2.2. Extraction of Region-of-Interest (ROI) and Image Preprocessing

**Figure 2:**
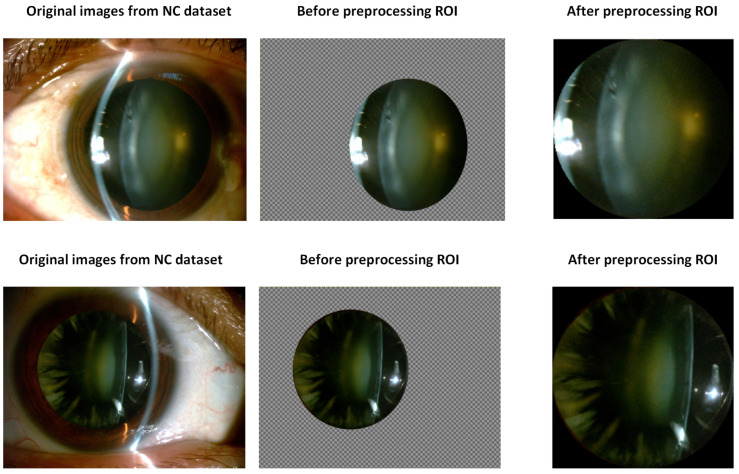
Example region of interest (ROI) extracted from the Nuclear Cataract Database, shown before and after preprocessing.

Manual extraction of the pupil region was performed, resulting in images containing a cropped pupil region and a transparent background, which allowed for precise rescaling of the ROI in the subsequent stage. The transparency had to be addressed by applying alpha channel masking to identify the visible pixels. Then, images were tightly cropped to the bounding box of the visible region, ensuring proper centering. Using area interpolation, they were resized to 224×224. The images before cropping, after extracting the ROI area, and after applying the described preprocessing are shown in Figure 2. Finally, they were converted to a PIL (Python Imaging Library) v10.1.0 image compatible with the neural network model. This process reduced the influence of background structures, enhancing the significant features of cataract.

### 2.3. Anomaly Detection Using an Autoencoder

There are many methods used in anomaly detection for one-class classification, such as One-class SVM [26], Plane-based One-Class Support Vector Machines [27], or hybrid models combined with neural networks that are used for categorizing OCT images anomalies [28], as well as various types of encoders [29].

An autoencoder is an unsupervised deep learning model that learns a data representation by compressing and reconstructing it [30]. It consists of two main components: an encoder, which transforms the input data into a hidden (latent) space, and a decoder, which reconstructs the data from this space back to a form similar to the original. Autoencoders are widely used in tasks such as anomaly detection in medical imaging [31,32].

Variational Autoencoders (VAEs) represent a generative extension of classical autoencoders [33]. They model data as a probability distribution in the latent space, allowing new data generation by sampling from this distribution. A Deep VAE typically consists of several layers in the encoder and decoder and utilizes ReLU activation functions [30]. In the context of semi-supervised anomaly detection, such a model is trained exclusively on “normal” examples, treating anomalies as deviations from a learned representation [30].

A Convolutional Autoencoder (CAE) is a type of autoencoder that uses convolutional layers. This makes it better suited for processing image data, preserving structural and spatial information [34]. A CAE enables more accurate image reconstruction, which is crucial in tasks where structural details—such as edges, textures, or subtle anatomical differences—play a significant role.

Slit-lamp photographs contain numerous fine, spatially distributed features, including nuclear coloration, subtle opalescence, and cortical micro-opacities. To effectively capture such clinically relevant details, a model architecture is required that can both extract key representations and preserve the spatial structure of the image.

A CAE meets these criteria more effectively than traditional SVM classifiers or a VAE. The use of 3 × 3 convolutional filters enables the CAE to learn local textures and edges, while its compact architecture with relatively few trainable parameters lowers the risk of overfitting, particularly in small medical datasets [34].

By contrast, a VAE generates reconstructions by sampling from Gaussian distributions, which often leads to blurring of complex anatomical structures and an increase in mean squared error (MSE), regardless of the presence of anomalies, thereby reducing classification performance. Similarly, one-class SVMs do not account for spatial pixel configurations, unlike CAEs, which can exploit local structural dependencies critical for detecting the morphological features of cataract in clinical images. This may result in the loss of diagnostically significant patterns and increase the likelihood of misclassification.

### 2.4. Tested Neural Network Architectures for LOCS III

The experiments were carried out on a standard PC with a Windows operating system with an Intel Core i7-11800H 2.3 GHz CPU, 16 GB of RAM, and an NVIDIA GeForce RTX 3060 Laptop GPU with 6 GB of memory. The training was performed for 100 epochs using the Cross-Entropy loss and optimized using Adam (learning rate = 0.0001). A ReduceLROnPlateau scheduler (patience = 2) was applied to adapt the learning rate.

To select the optimal architecture and evaluate classification performance, the dataset was tested using six different neural network models, each differing in structure, number of parameters, and intended application.

The VGG16 architecture is characterized by a deep 16-layer structure. It consists of small 3×3 convolutional filters followed by pooling layers and concludes with fully connected layers. It has a large number of parameters, 138 million [35].

ResNet50 is a deep convolutional neural network with 50 layers that utilizes residual learning through shortcut connections. This architecture employs bottleneck residual blocks consisting of 1×1, 3×3 convolutions to effectively increase the depth while limiting the parameters [36].

VGG11 is a lightweight variant of the VGG16 architecture, comprising alternating convolutional and pooling layers for effective feature extraction. However, it generally contains a more significant number of parameters than modern architectures [35].

ResNet18, a compact variant of the ResNet architecture, utilizes residual connections to address the issue of vanishing gradients in deep networks. This design improves its suitability for medical image classification, achieving high accuracy, while maintaining a low computational complexity [36].

MobileNetV3 is optimized for mobile devices, featuring a low parameter count and depthwise separable convolutions, as well as inverted residual blocks, for efficient classification on limited hardware [37].

EfficientNet is a family of models ranging from B0 to B7, designed for optimal performance. It uses compound scaling to balance network depth and input resolution. It can achieve high classification accuracy with fewer parameters and a lower computational cost [38].

The trained neural network model was saved in the .pth format from PyTorch (v2.5.1). It was first exported to the ONNX (v1.14.0) format, then converted to a TensorFlow (v2.13.0) model, and finally transformed into the .tflite format for compatibility with mobile devices.

### 2.5. Android-Based Application

**Figure 3:**
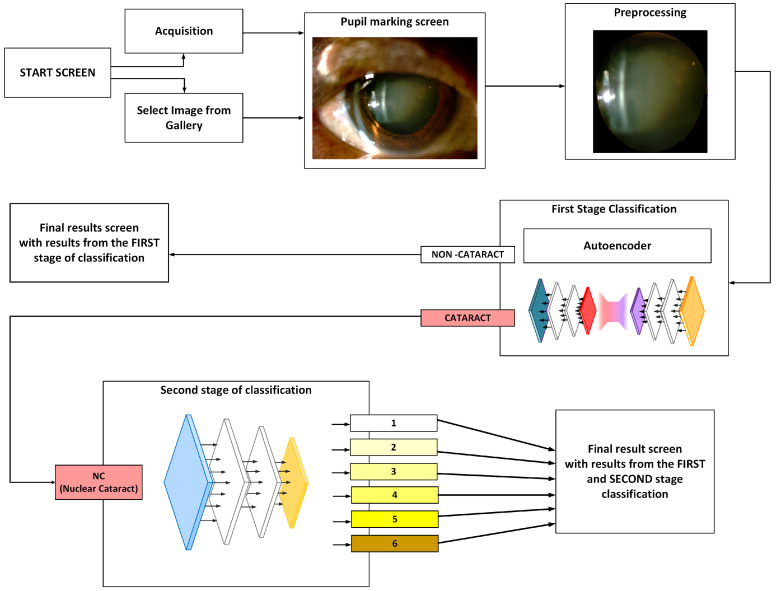
Mobile application architecture overview.

The cataract classification was implemented in a mobile application for Android phones. The development was performed in Android Studio version 2025.1.1 and the Kotlin programming language. A diagram of the application is shown in Figure 3.

After selecting an image, it undergoes preprocessing and passes through an initial binary classification step. If the image is classified as belonging to the CATARACT category, it is further analyzed using a deep neural network, which assigns it to one of six classes following the LOCS III cataract grading system.

**Figure 4:**
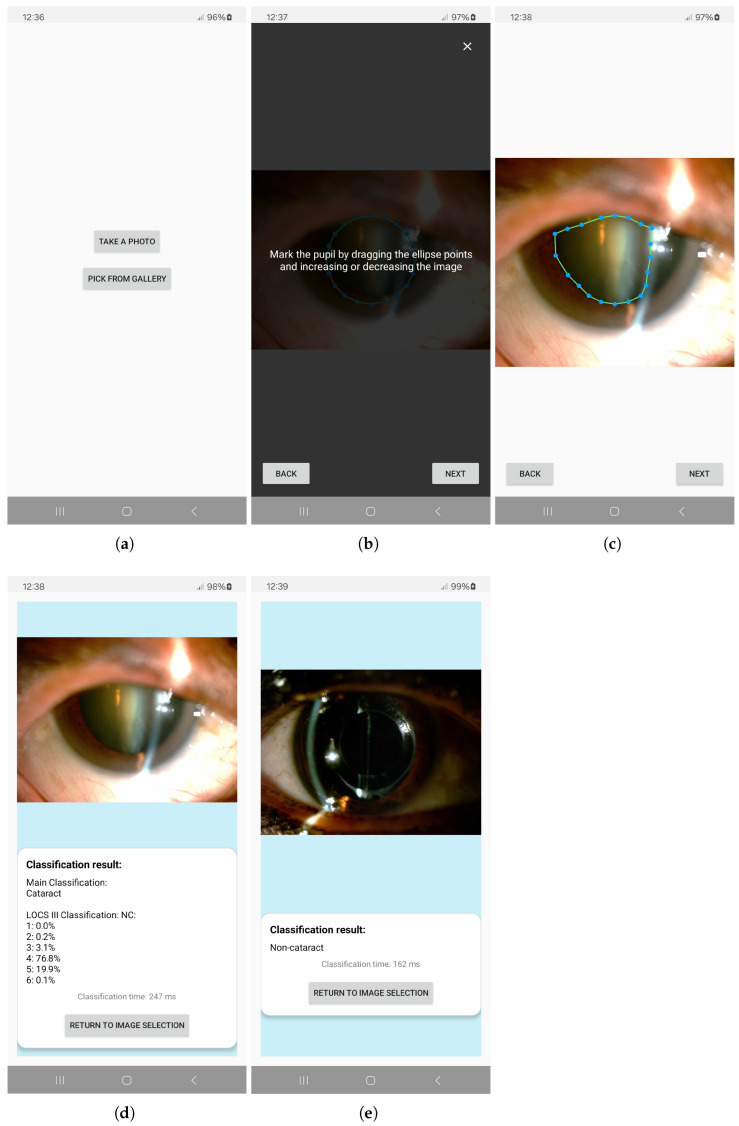
(**a**) Screen for selection between using an image from the gallery of capturing a new one, (**b**) before marking the pupil, with information on how to do it, (**c**) after marking pupil, customizing the detection circle, (**d**) screen displaying the classification results for both stages of classification, as the image was classified as cataract, (**e**) screen displaying the classification results for only the first stage of classification, as the image was classified as non-cataract.

Upon initialization of the application, the user is presented with an introductory interface. By interacting with this interface, the user navigates to a subsequent screen that facilitates the selection of one of two actions: capturing a new image using the device’s camera or selecting an existing image from the device’s gallery, as shown in Figure 4a.

In the case when the user selects an image from the gallery, an additional interface is displayed, providing functionality for zooming in and out of the image and for delineating the pupil region using a customizable, circular contour (Figure 4b,c).

The image is cropped to retain only the selected region of interest (ROI), which is then subjected to classification. During the analysis process, a loading screen is presented to the user. Upon completion of the classification, the interface displays the original image alongside the corresponding classification result (Figure 4d,e).

When the user opts to capture a new image, they are given the option to select either the front-facing or rear-facing camera of the device. Following image acquisition, the user is once again allowed to adjust the region of interest (ROI) accordingly, after which the image is subjected to classification.

## 3. Results

### 3.1. Dataset

This study used images from the Nuclear Cataract Database [24] to train and assess the effectiveness and accuracy of the proposed mobile solution that employed deep learning and artificial neural networks to detect and classify nuclear cataract grade. To achieve the highest possible accuracy for the neural network models, images classified from 1 to 6 for NC grade, consistent with the LOCS III scale, were used for training and evaluation. Considering insufficient cataract visibility, image blurriness, or poor illumination of the pathological area, an ophthalmology specialist selected images that accurately represented the degrees of advancement. The number of images chosen for each class is written in Table 1. A detailed list of selected files is provided by the authors in the GitHub repository: https://github.com/ignatowicz-alicja/AI-Powered-Mobile-App-for-Nuclear-Cataract-Detection (accessed on 19 May 2025).

### 3.2. Anomaly Detection

The first stage of classification involved binary classification of the images into cataract and non-cataract categories. Since the Nuclear Cataract Database does not contain images of healthy eyes, and there are no publicly available slit-lamp image datasets with a distinct class, we employed a convolutional autoencoder in our experiments. To test the model’s accuracy, images from the Nuclear Cataract database classified as IOL were used for the non-cataract class. This IOL class features images of eyes after cataract surgery, where the cloudy natural lens has been removed and replaced with a clear intraocular lens.

**Figure 5:**
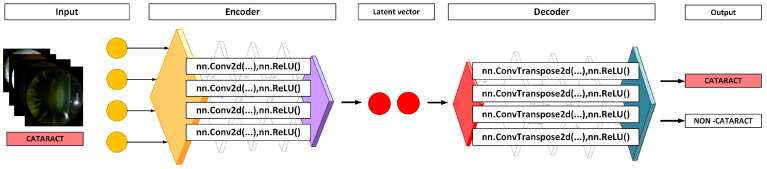
Diagram presenting the sequential steps involved in the binary classification process.

Before implementing the autoencoder, the data underwent preprocessing, as outlined in the previous section. The autoencoder model features a convolutional encoder that consists of four convolutional layers, each followed by a ReLU activation function. Similarly, the decoder is composed of four deconvolutional layers, with each layer also incorporating a ReLU activation function (Figure 5).

**Figure 6:**
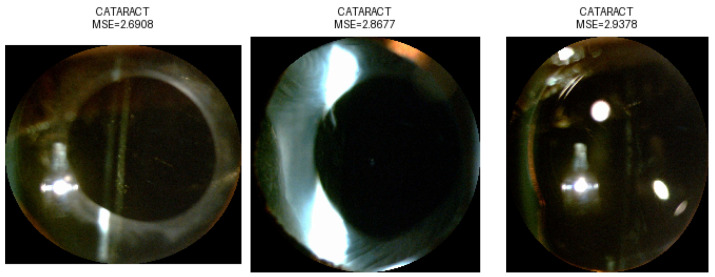
False classification of IOL images as CATARACT.

**Figure 7:**
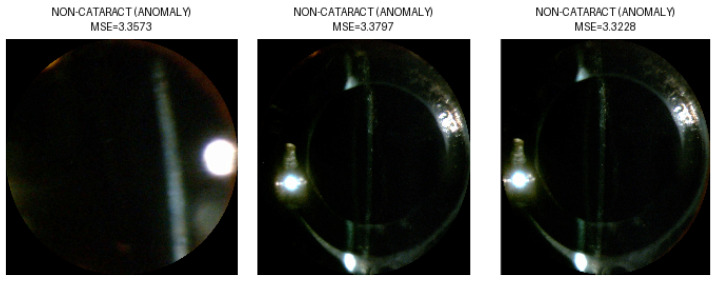
Correct classification IOL images as NON-CATARACT.

**Figure 8:**
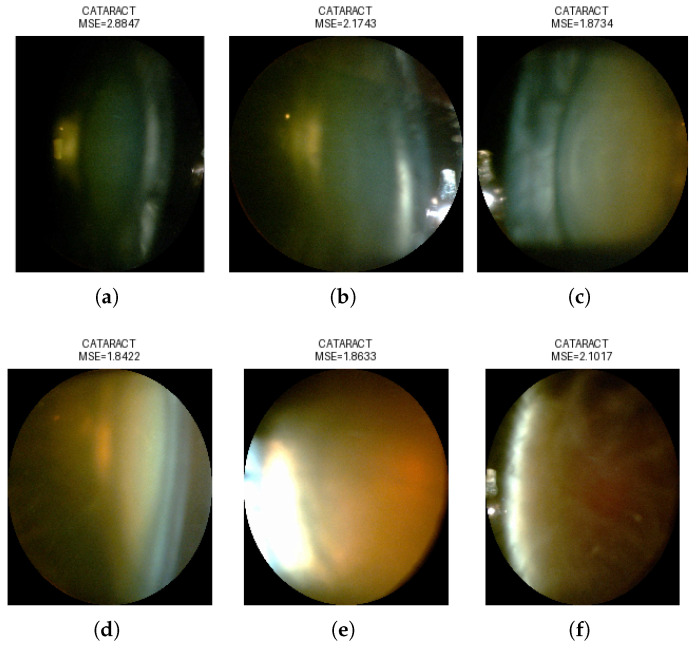
Correct classification from images NC class as CATARACT: (**a**) class 1 (**b**) class 2 (**c**) class 3 (**d**) class 4 (**e**) class 5 (**f**) class 6.

**Figure 9:**
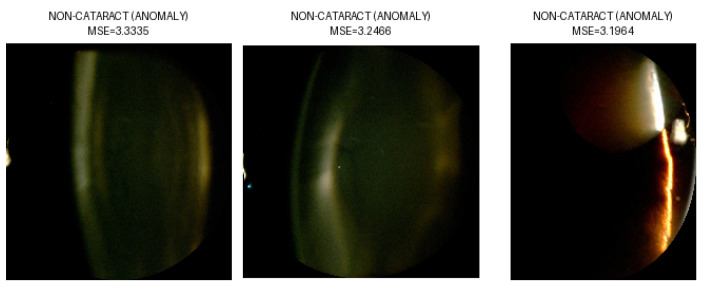
False classification of images from NC class as NON-CATARACT.

Mean Squared Error (MSE) assessed the autoencoder’s image reconstruction quality by measuring the average squared difference between original and reconstructed pixel values. Reversed anomaly detection logic was applied, due to the lack of available healthy eye images; images of eyes with cataracts were considered the “NORMAL” class. Due to the high variability in the images, a strict classification threshold was applied. This was determined based on the 1st percentile of the reconstruction error distribution. This threshold captured the most accurately reconstructed images, enabling a high classification accuracy of 94.6% tested on all selected images—as cataract class—and images from class IOL—as non-cataract class. Examples of the performed classification are presented in Figure 6, Figure 7, Figure 8 and Figure 9, illustrating both correctly classified images and misclassified cases.

### 3.3. Selection of Neural Network for Second Stage of Classification

The selected dataset was split into a 80% training set, 10% validation set, and 10% testing set. Additionally, to ensure the network learned from all classes, the dataset splitting was stratified, thereby preserving the class proportions within each subset.

Furthermore, data augmentation techniques were employed, including random horizontal flip with a probability of 0.5, random rotation from −15 to +15 degrees, and color jitter randomly adjusting brightness and contrast by a factor of up to 0.2. Subsequently, the images were converted to tensors and normalized using ImageNet statistics. These operations increased the dataset diversity and expanded its size to 1272 images. The classification performance is presented in Table 2.

**Figure 10:**
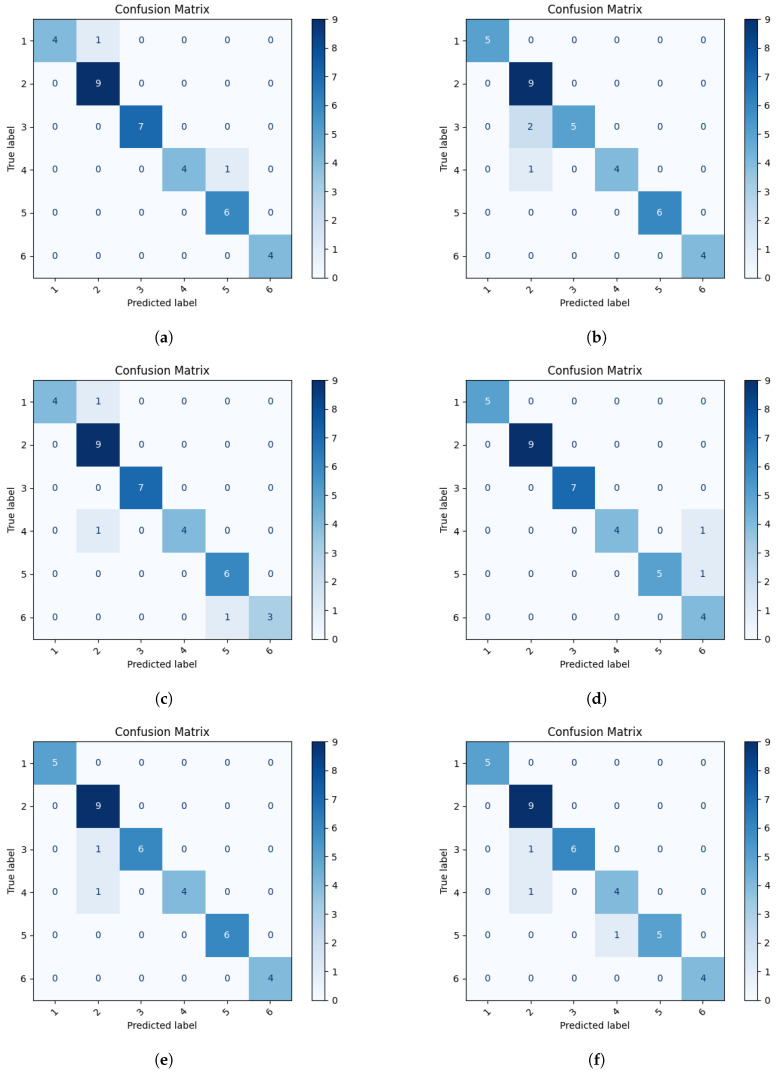
Confusion matrices for the first stage of classification using: (**a**) VGG16 (**b**) ResNet50 (**c**) VGG11 (**d**) ResNet18 (**e**) MobileNetV3 (**f**) EfficientNet.

Among the full-scale models, VGG16 demonstrated the best performance, surpassing ResNet50 in terms of both accuracy and other classification quality metrics. ResNet50 achieved an accuracy of 91.3% and an F1-score of 93.2%. In comparison, VGG16 attained an accuracy of 94.4%, with an F1-score of 94.1% indicating its higher stability in recognizing images across all classes. Despite comparable metric values, ResNet50 may exhibit reduced stability in classifying less representative classes, as shown in the confusion matrix on Figure 10b, where the model misclassified images belonging to classes 3 and 4, classes particularly challenging to due to their high visual similarity.

The lighter neural network architectures performed comparably well, in some cases even surpassing the full-scale models. The VGG11 model achieved an accuracy of 91.6%, with classification quality metrics similar to those obtained by ResNet50. ResNet18 achieved a high classification accuracy of 94.5% and an F1-score of 95.2%, demonstrating its high effectiveness and stability. MobileNetV3 also exhibited excellent performance, achieving an accuracy of 94.2% and metric values surpassing those of ResNet18. However, both ResNet18 and MobileNetV3 exhibited classification errors for images belonging to grade 4 (Figure 10d,e).

The efficientNet model achieved a slightly lower accuracy (91.1%) and an F1-score of 91.3%. Furthermore, it demonstrated a higher number of misclassifications for images in grades 3, 4, and 5 (Figure 10f).

All analyzed neural network architectures achieved classification accuracies exceeding 90%, confirming their high efficacy in the task of nuclear cataract (NC) grading. The ResNet18 and VGG16 models achieved the highest accuracy rates. This makes them strong choices for use in mobile applications. The accuracy of most models significantly declined after conversion to the TensorFlow Lite format, which is designed for mobile device deployment. While the models achieved accuracies of 91–95% in their original training environment, their performance frequently dropped to below 80%, and in some cases even below 70%, after conversion.

Converting a model to TensorFlow Lite format introduces simplifications and optimizations that can alter the behavior of certain layers. The optimized TFLite inference runtime may also lead to subtle numerical discrepancies in floating-point computations. Furthermore, if the model contains operations not natively supported by TFLite, these are either approximated or delegated to custom implementations, which can potentially degrade accuracy (Table 3). The most significant drops were observed for MobileNetV3 and EfficientNet, with their accuracies decreasing to approximately 63%. ResNet18, despite its good pre-conversion results, achieved only 68% accuracy after transformation. VGG11 and ResNet50 exhibited a smaller decline in performance, dropping to about 84% and 82%.

VGG11 achieved the best classification quality metrics and highest accuracy in the TFLite format among all analyzed models. Consequently, this model was selected and implemented in the mobile application for nuclear cataract classification. To perform an additional check for possible overfitting before converting the VGG11 model, a 10-fold cross-validation experiment was conducted to assess the risk of overfitting, using the same training parameters as described in the manuscript. Data augmentation increased the size of the dataset to 1272 images. The mean training accuracy achieved across all folds was 96.2%, while the mean validation accuracy reached 91.6%. This difference of 4.6 percentage points falls well below the commonly accepted threshold of 10 percentage points above which overfitting is typically suspected.

The standard deviation of this difference was 4.7 percentage points, with fold-specific values ranging from 1.2 to 7.1 percentage points. Importantly, none of the validation folds exceeded the 10-point threshold. These findings indicate that the model did not exhibit signs of overfitting and that its performance was consistent and stable across different data partitions.

### 3.4. App Performance on Mobile Devices

**Figure 11:**
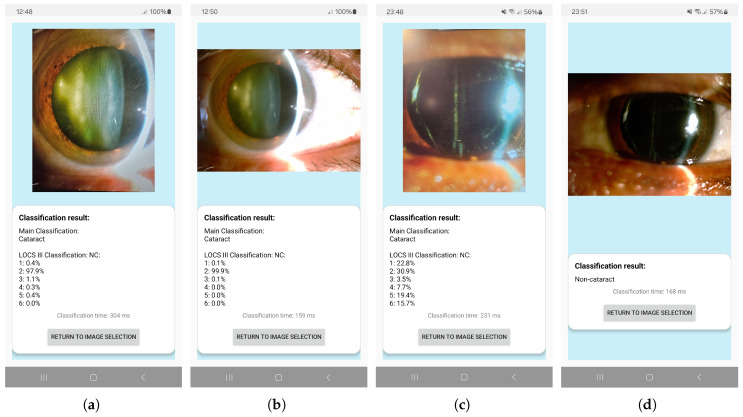
Classification results for the same image from dataset from class 2 (**a**), correctly classified picture taken after selecting the ‘Take a picture’ option (**b**), correctly classified picture picked from the mobile device gallery. Classification results for the same image from dataset form class IOL, (**c**) falsely classified picture taken after selecting the ‘Take a picture’ option, (**d**) correctly classified picture chosen from the mobile device gallery.

The experimental results showed that the average classification time for gallery images was 162.4 ms, and images captured using the camera required 186.5 ms on average. Images captured with the smartphone camera were more likely to be misclassified in the first stage of classification. Especially in cases as in Figure 11c, where overexposure occurred in the pupil region, caused by uneven or excessive lighting. This pixel saturation in ROI led to distortion in the feature space used to calculate the MSE between the feature vector and the typical representation of cataract in images. Consequently, the model was more likely to produce false positives, misclassifying an image as cataract even when non-cataract-related features were present.

These observations clearly indicate that image quality and acquisition conditions played a critical role in the classification accuracy. Degraded image quality—due to overexposure, blur, noise, or improper framing—could significantly impair the classifier’s ability to make reliable predictions. As part of future development, we plan to implement real-time quality assessment mechanisms to alert the user to poor image quality, as well as automated exposure correction and contrast enhancement algorithms to mitigate the effects of acquisition artifacts.

## 4. Conclusions

Artificial intelligence in ophthalmology can indeed assist in interpreting medical data and can be used for screening tests, serving as a digital second opinion; however, the final decision is always up to the doctor. Modern smartphones are capable of running such AI-based applications due to having sufficient computing power. It should be noted that the simplified architectures operating on smartphones using TensorFlow Lite did not significantly reduce the values of the quality metrics of the classification process.

The presented application, available in open-source form on github, can be further developed. The user interface for the cataract classification process shows decision probabilities for the LOCS III standard. This solution could be expanded to other types of cataracts. The key element is to prepare an appropriate amount of training data to obtain a model with high accuracy metrics.

## Figures and Tables

**Table 1:** Number of images from the Nuclear Cataract Database compared to the number of images used in the experiment.

Images from the NC Dataset	NC						Total
	1	2	3	4	5	6	
Original	54	247	129	93	70	34	627
Selected	42	90	63	49	58	33	335

**Table 2:** Classification performance for network architectures.

Stage	Classification	Network Architecture	Accuracy	Precision	Recall	F1-Score
Stage II: NC	1, 2, 3, 4, 5, 6	VGG16	0.944	0.962	0.934	0.941
		ResNet50	0.913	0.945	0.921	0.932
		VGG11	0.916	0.937	0.914	0.918
		ResNet18	0.945	0.963	0.940	0.952
		MobileNetV3	0.942	0.974	0.945	0.955
		EfficiencyNet	0.911	0.931	0.916	0.913

**Table 3:** Performance of neural network models after conversion to TensorFlow Lite.

Neural Network	Accuracy	Precision	Recall	F1-Score
VGG16	0.821	0.831	0.820	0.820
ResNet50	0.824	0.852	0.823	0.824
VGG11	0.848	0.857	0.848	0.846
ResNet18	0.681	0.784	0.680	0.658
MobileNetV3	0.633	0.737	0.632	0.615
EfficientNet	0.638	0.698	0.612	0.669

## Data Availability

The Kotlin source code and a ready-to-download application have been made available under the link https://github.com/ignatowicz-alicja/AI-Powered-Mobile-App-for-Nuclear-Cataract-Detection (accessed on 19 June 2025). A list of selected images from the publicly available database used in all experiments has also been provided on GitHub.

## Abbreviations

The following abbreviations are used in this manuscript:

CAE	Convolutional Autoencoder
IOL	Intraocular Lens
LOCS III	Lens Opacities Classification System III
MSE	Mean Squared Error
NC	Nuclear Cataract
PIL	Python Imaging Library
ROI	Region of Interest
SVM	Support Vector Machines
